# Bioinformatics analysis and prediction of Alzheimer’s disease and alcohol dependence based on Ferroptosis-related genes

**DOI:** 10.3389/fnagi.2023.1201142

**Published:** 2023-07-13

**Authors:** Mei Tian, Jing Shen, Zhiqiang Qi, Yu Feng, Peidi Fang

**Affiliations:** ^1^The Affiliated Jiangsu Shengze Hospital of Nanjing Medical University, Nanjing, China; ^2^Medicine and Health, The University of New South Wales, Kensington, NSW, Australia; ^3^Melbourne Medical School, The University of Melbourne, Parkville, VIC, Australia

**Keywords:** Alzheimer’s disease, alcohol dependence, Ferroptosis, cybB, STEAP3, ACSL4

## Abstract

**Background:**

Alzheimer’s disease (AD) is a neurodegenerative disease whose origins have not been universally accepted. Numerous studies have demonstrated the relationship between AD and alcohol dependence; however, few studies have combined the origins of AD, alcohol dependence, and programmed cell death (PCD) to analyze the mechanistic relationship between the development of this pair of diseases. We demonstrated in previous studies the relationship between psychiatric disorders and PCD, and in the same concerning neurodegeneration-related AD, we found an interesting link with the Ferroptosis pathway. In the present study, we explored the bioinformatic interactions between AD, alcohol dependence, and Ferroptosis and tried to elucidate and predict the development of AD from this aspect.

**Methods:**

We selected the Alzheimer’s disease dataset GSE118553 and alcohol dependence dataset GSE44456 from the Gene Expression Omnibus (GEO) database. Ferroptosis-related genes were gathered through Gene Set Enrichment Analysis (GSEA), Kyoto Encyclopedia of Genes and Genomes (KEGG), and relevant literature, resulting in a total of 88 related genes. For the AD and alcohol dependence datasets, we conducted Limma analysis to identify differentially expressed genes (DEGs) and performed functional enrichment analysis on the intersection set. Furthermore, we used ferroptosis-related genes and the DEGs to perform machine learning crossover analysis, employing Least Absolute Shrinkage and Selection Operator (LASSO) regression to identify candidate immune-related central genes. This analysis was also used to construct protein-protein interaction networks (PPI) and artificial neural networks (ANN), as well as to plot receiver operating characteristic (ROC) curves for diagnosing AD and alcohol dependence. We analyzed immune cell infiltration to explore the role of immune cell dysregulation in AD. Subsequently, we conducted consensus clustering analysis of AD using three relevant candidate gene models and examined the immune microenvironment and functional pathways between different subgroups. Finally, we generated a network of gene-gene interactions and miRNA-gene interactions using Networkanalyst.

**Results:**

The crossover of AD and alcohol dependence DEG contains 278 genes, and functional enrichment analysis showed that both AD and alcohol dependence were strongly correlated with Ferroptosis, and then crossed them with Ferroptosis-related genes to obtain seven genes. Three candidate genes were finally identified by machine learning to build a diagnostic prediction model. After validation by ANN and PPI analysis, ROC curves were plotted to assess the diagnostic value of AD and alcohol dependence. The results showed a high diagnostic value of the predictive model. In the immune infiltration analysis, functional metabolism and immune microenvironment of AD patients were significantly associated with Ferroptosis. Finally, analysis of target genes and miRNA-gene interaction networks showed that hsa-mir-34a-5p and has-mir-106b-5p could simultaneously regulate the expression of both CYBB and ACSL4.

**Conclusion:**

We obtained a diagnostic prediction model with good effect by comprehensive analysis, and validation of ROC in AD and alcohol dependence data sets showed good diagnostic, predictive value for both AD (AUC 0. 75, CI 0.91–0.60), and alcohol dependence (AUC 0.81, CI 0.95–0.68). In the consensus clustering grouping, we identified variability in the metabolic and immune microenvironment between subgroups as a likely cause of the different prognosis, which was all related to Ferroptosis function. Finally, we discovered that hsa-mir-34a-5p and has-mir-106b-5p could simultaneously regulate the expression of both CYBB and ACSL4.

## 1. Introduction

The global incidence of dementia has almost tripled in recent decades, rising from 20.2 million cases in 1990 to 57.4 million in 2019 (GBD 2019 Dementia Forecasting Collaborators, 2022). It is projected that by 2050, the number of individuals with dementia worldwide will reach 152 million (Li, 2018).

Alzheimer’s disease (AD), a neurodegenerative disorder, is marked by amyloid plaques, tau protein tangles, and brain atrophy (Ausó et al., 2020). AD, along with vascular dementia, constitutes the majority of dementia cases. Cognitive decline in later life has been linked to long-term alcohol consumption, AD, and vascular dementia (Downs et al., 2023; Marsland et al., 2023).

In 2020, The Lancet Commission’s report identified excessive or harmful alcohol consumption in midlife as one of the major modifiable risk factor for dementia (Livingston et al., 2020). This finding is reinforced by a wealth of evidence demonstrating the neurotoxic effects of ethanol on the brain, which can lead to structural and functional alterations that impair cognitive function (Topiwala and Ebmeier, 2018; Rao and Topiwala, 2020). Notably, alcohol-induced neurotoxicity can exacerbate the progression of neurodegenerative diseases like AD and vascular dementia.

Ferroptosis is an iron- and lipid peroxidation-dependent form of cell death that has been increasingly linked to a variety of neurodegenerative diseases, such as AD, motor neuron disease, Parkinson’s disease, Huntington’s disease, and Friedreich’s ataxia (FRDA) (Lane et al., 2021).

Dixon et al. (2012) first reported and named the iron-dependent mode of cell death Ferroptosis. Ferroptosis differs from apoptosis, necroptosis, and other forms of cell death in that it is caused by the iron-dependent accumulation of lipid peroxides (Costa et al., 2023). Biochemically, it is characterized by glutathione (GSH) depletion, reduced GPX4 activity, and increased ROS production through the Fenton reaction (Li et al., 2020). Furthermore, NTN-1 treatment enhanced the expression of PPARγ, nuclear factor erythroid 2-related factor 2 (Nrf2), and glutathione peroxidase 4 (GPX4), which are essential regulators of ferroptosis in EBI after SAH (Chen J. et al., 2023).

The studies about whether the Ferroptosis pathway is linked to alcohol dependence and AD and the exact mechanism of their relationship were absent. We demonstrated the link between psychiatric disorders and neurodegenerative changes with PCD and mitochondrial function in our earlier studies, and the present study is a tentative exploration of Ferroptosis, AD, and alcohol dependence from a bioinformatic genetic perspective. The difference in immunological microenvironment may be one of the causes of the varied prognoses, which is related to the function of ferroptosis, finally, we obtained a good diagnostic prediction model.

## 2. Materials and methods

### 2.1. Materials

**FIGURE 1:**
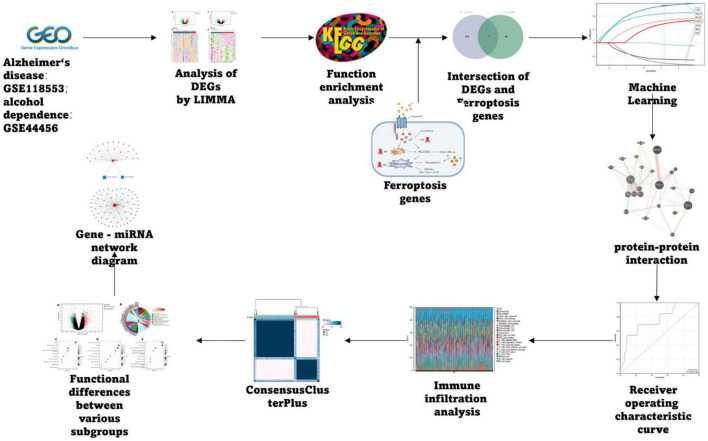
Flow chart.

The AD dataset GSE118553 and alcohol dependence dataset GSE44456 were chosen from the GEO database^1^ (Barrett et al., 2013); Ferroptosis-related genes were collected through the Kyoto Encyclopedia of Genes and Genomes (KEGG), Gene Set Enrichment Analysis (GSEA), and related literature, and finally, 88 related genes were obtained, and the specific process is depicted in Figure 1.

### 2.2. Determination of DEGs

Linear models for microarray data (Limma) (Sokhansanj et al., 2004) is a generalized linear regression model that provides differential expression screening methodology for determinations of DEGs. In this study, R-package-based limma (version 3.40.6) was used for differential analysis to obtain DEGs between various comparison groups and controls. Here we used | log2 fold change (FC)| > 1 and a *P*-value less than 0.05 as the condition for determining DEGs by the Limma package. Further, the heat and volcano maps of DEGs for AD and alcohol dependence were visualized by sangerBox, respectively (Shen et al., 2022).

### 2.3. Gene function enrichment analysis

We cross-screened the above-processed DEGs in AD and those in alcohol dependence using the Venn diagram to find the genes related to AD and alcohol dependence for Gene Set Enrichment Analysis (GSEA). To perform GSEA, the latest gene annotations of the KEGG Pathway were obtained using the KEGG rest API.^2^ The GO annotations in the R-package^3^ (v. 3.1.0) were utilized as the background to plot genes to the background set. R-package, known as clusterProfiler (v 3.14.3) (Yu et al., 2012), was employed for enrichment analysis for obtaining the GSEA results. Based on gene expression profiles and phenotypic groupings, we kept the minimum gene set at five and the maximum at 5,000. A *p*-value of less than 0.05 and an FDR of less than 0.1 were deemed statistically significant.

### 2.4. Machine learning identification of candidate genes associated with Ferroptosis in Alzheimer’s disease combined with alcohol dependence

The R-package, glmnet (Zhang et al., 2019), and RandomForest (Yasir et al., 2022) were used to integrate data related to survival status, survival time, and gene expression for regression analysis utilizing lasso-cox and Random Forest methods. Additionally, 10-fold cross-validation was set up to attain the optimal model. We cross-screened the results of the two kinds of machine learning by using a Venn diagram to obtain the final diagnostic prediction model.

### 2.5. Protein-protein interaction network construction

The protein-protein interaction networks (PPI) were constructed with the help of the GeneMANIA database. The latter is a user-friendly and flexible website for hypotheses generation regarding gene lists analysis, gene function, and prioritization of genes for functional analysis (Franz et al., 2018).

### 2.6. Diagnostic model validation

We utilized pROC (Robin et al., 2011) in the R-package to perform Receiver Operating Characteristic (ROC) analysis to generate the Area Under the Curve (AUC). It was also used to evaluate AUC and confidence intervals (CIs) with the help of the CI function of pROC for obtaining the final AUC results. These results were visualized using sangerBox. The whole method helped to observe the expression of the characteristic genes in the AD dataset GSE118553 and the alcohol dependence dataset GSE44456.

### 2.7. Subgroup analysis by candidate genes

Cluster analysis was performed using ConsensusClusterPlus (Wilkerson and Hayes, 2010), using agglomerative pam clustering with a 1-spearman correlation distances and resampling 80% of the samples for 10 repetitions. The optimal number of clusters was determined using the empirical cumulative distribution function plot.

Unsupervised hierarchical cluster analysis was performed on IS samples using R’s “ConsensusClusterPlus” (Wilkerson and Hayes, 2010) and the gene expression of candidate genes as input information. The different subgroups were subjected to Limma analysis to obtain subgroup DEGs, and functional differences between subgroups were analyzed by KEGG and GO.

### 2.8. Analysis of immune microenvironment

Immuno-oncology biological research (IOBR) (Zeng et al., 2021) is a computational tool utilized in immuno-oncology biology studies. In this study, the CIBERSORT (Newman et al., 2015) method was selected on the basis of our expression profiles using the R-package IOBR to calculate the 22 immune infiltrating cell scores for each sample. Analysis of immune cell infiltration was conducted by Cibersort in the R-package, and its correlation was calculated using the spearman coefficient, and a heat map of infiltrating immune cell correlation was performed using corrplot in the R-package.

### 2.9. Hub genes-miRNA prediction

To predict the miRNAs of candidate genes, we created a gene-miRNA interaction network through Networkanalyst^4^ (Zhou et al., 2019).

## 3. Results

### 3.1. Determination of DEGs in Alzheimer’s disease and alcohol dependence

**FIGURE 2:**
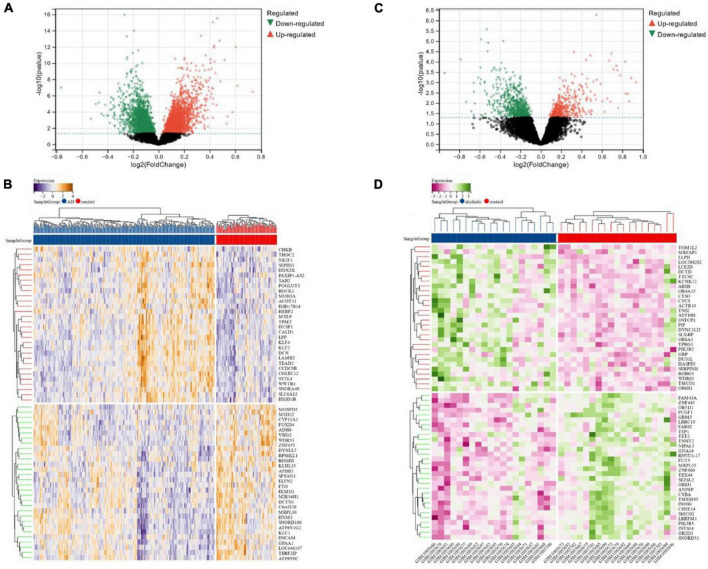
**(A,B)** Volcano maps and heat maps of DEGs in Alzheimer’s disease; **(C,D)** volcano and heat maps of differentially expressed genes in alcohol-dependent.

We identified a total of 6,023 DEGs in the dataset corresponding to AD (GSE118553) utilizing the Limma method, out of which 2,745 were down-regulated and 3,278 were up-regulated (Figures 2A, B). In total, 1,101 DEGs were screened in the alcohol dependence dataset (GSE44456), of which 658 were down-regulated and 443 were up-regulated (Figures 2C, D).

### 3.2. FEA of candidate genes linked with Alzheimer’s disease and alcohol dependence

**FIGURE 3:**
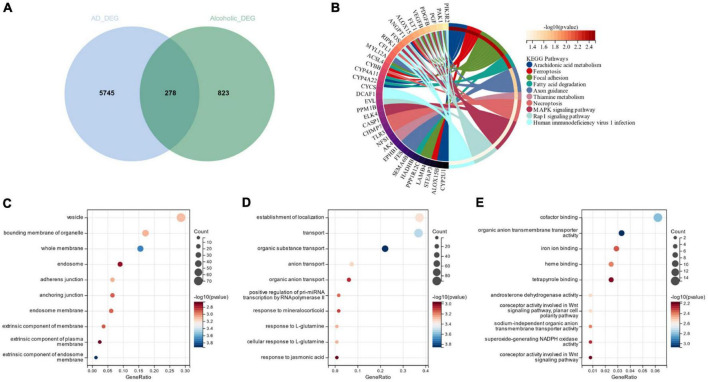
**(A)** Venn diagram of candidate genes linked with Alzheimer’s disease and alcohol dependence; **(B)** Candidate gene KEGG analysis; **(C)** Candidate genes GO analysis for cellular composition; **(D)** Candidate genes GO analysis for biological processes; **(E)** Candidate genes GO analysis for molecular function.

Figure 3A depicts the 278 cross-selected candidate genes related to AD and alcohol dependence in a Venn diagram functional enrichment analysis (FEA) was conducted on the candidate genes, and KEGG analysis depicted that the candidate genes were primarily enriched in “Arachidonic acid metabolism,” “Focal adhesion” and “Ferroptosis” pathways (Figure 3B). We observed that both AD and alcohol dependence are closely associated with Ferroptosis. GO analysis revealed that in terms of cellular components, candidate genes were chiefly located in “vesicle,” “bounding membrane of organelle” and “whole membrane” (Figure 3C). The primary biological processes of the candidate genes constitute “establishment of localization,” “transport” “organic substance transport” and other transport-related processes (Figure 3D). Molecular function (MF) depicted that the most crucial item among the candidate genes was “cofactor binding” (Figure 3E).

### 3.3. Identification of candidate genes linked with Ferroptosis in Alzheimer’s disease combined with alcohol dependence by machine learning and PPI network construction

**FIGURE 4:**
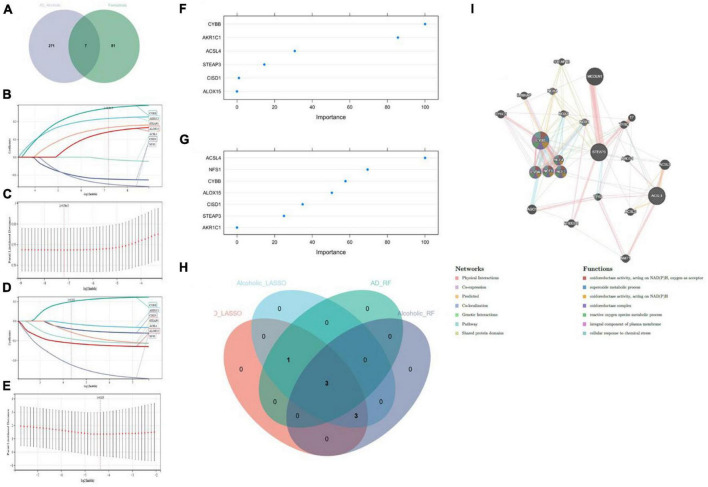
**(A)** Crossover Venn diagram of Alzheimer’s disease with alcohol dependence-related candidate genes and Ferroptosis-related genes; **(B,C)** LASSO regression candidate gene identification for Alzheimer’s disease dataset; **(D,E)** LASSO regression candidate gene identification for alcohol dependence dataset; **(F)** RF regression candidate gene identification for Alzheimer’s disease dataset; **(G)** RF regression candidate gene identification for alcohol dependence dataset; **(H)** machine learning Venn diagram cross-identification; **(I)** Candidate genes PPI network construction.

We cross-analyzed the AD with alcohol dependence-associated candidate genes and Ferroptosis-associated genes to obtain seven associated genes (Figure 4A). And LASSO regression was applied for candidate gene identification. Seven potential candidate genes were identified and determined in both the AD dataset and the alcohol dependence dataset (Figures 4B–E). We also used RF regression for the identification of candidate genes, from which four potential candidate biomarkers were determined in the AD dataset (Figure 4F), and six potential candidate biomarkers were determined in the alcohol dependence dataset (Figure 4G). The genes identified by these two-machine learning (ML) algorithms were then cross-analyzed, which resulted in three candidate genes (CYBB, STEAP3, and ACSL4) (Figure 4H). Based on these three candidate genes, the establishment of a PPI network was performed in which Physical Interactions made up 77.64%, and Co-expression made up 8.01%. Further, they were primarily involved in oxidoreductase activity and the superoxide metabolic process, as depicted in Figure 4I.

### 3.4. Diagnostic model validation

**FIGURE 5:**
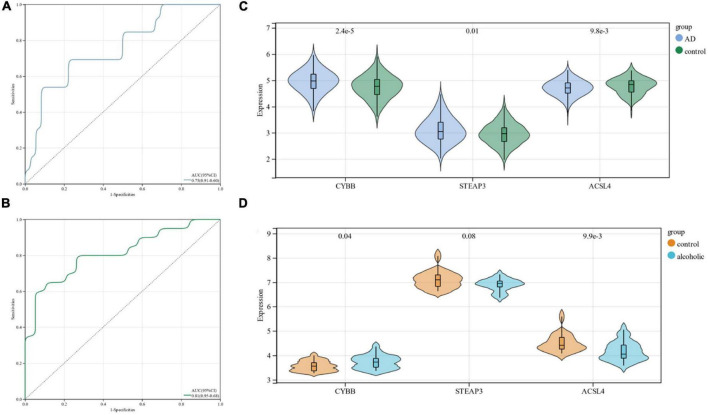
**(A)** ROC curves in the Alzheimer’s disease dataset; **(B)** ROC curves in the alcohol dependence dataset; **(C)** candidate gene expression profile analysis in the Alzheimer’s disease dataset; **(D)** candidate gene expression profile analysis in the alcohol dependence dataset.

Using ROC curves, we validated the diagnostic value of these three candidate genes at the stage when all candidate genes were used as joint indicators in the AD dataset (AUC 0. 75, CI 0.91–0.60) (Figure 5A). We also validated its diagnostic model into the alcohol dependence dataset and showed (AUC 0.81, CI 0.95–0.68) an excellent diagnostic significance (Figure 5B). Further, an expression profile analysis was conducted of the three candidate genes (Figures 5C, D). The results revealed that there were statistically significant differences (*p* < 0.05) among the candidate genes, except for STEAP3, which did not differ significantly in the alcohol dependence dataset.

### 3.5. Analysis of immune cell infiltration

Numerous studies have shown that alcohol consumption leads to excessive inflammation in vital organs including liver, intestines, and brain (Crews, 2012; Szabo et al., 2012; Szabo and Lippai, 2014).

Moreover, alcohol-induced innate immune activation in the central nervous system (CNS) has been shown to mediate neurotoxicity and ethanol-induced behaviors including alcohol addiction and cognitive decline in preclinical and clinical setting (Crews, 2008).

**FIGURE 6:**
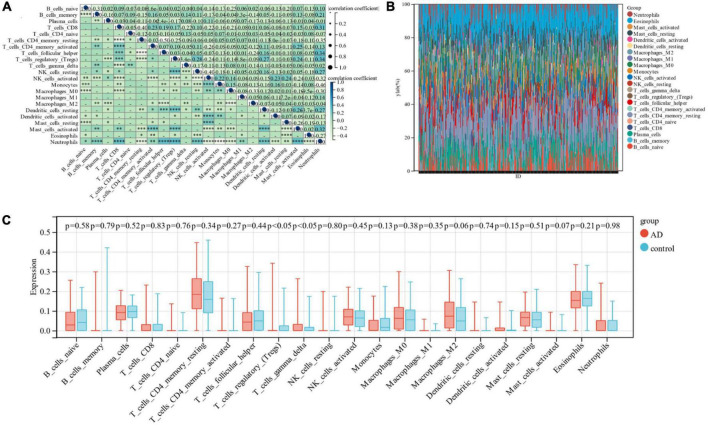
**(A)** Correlation between 22 immune cells; **(B)** relative proportion of 22 immune cells in each sample; **(C)** difference in immune infiltration between the Alzheimer’s sample group and the control sample.

In this research, the Cibersort algorithm was utilized to estimate the proportion of 22 immune cells in Alzheimer’s samples and control samples (Figures 6A, B). We compared the immune cell infiltration in Alzheimer’s samples and control samples using box line plots (Figure 6C). The results depicted a significant difference between the two groups in Regulatory T cells and Gamma delta T cells (*p* < 0.05).

### 3.6. Consensus clustering analysis of candidate gene clusters

**FIGURE 7:**
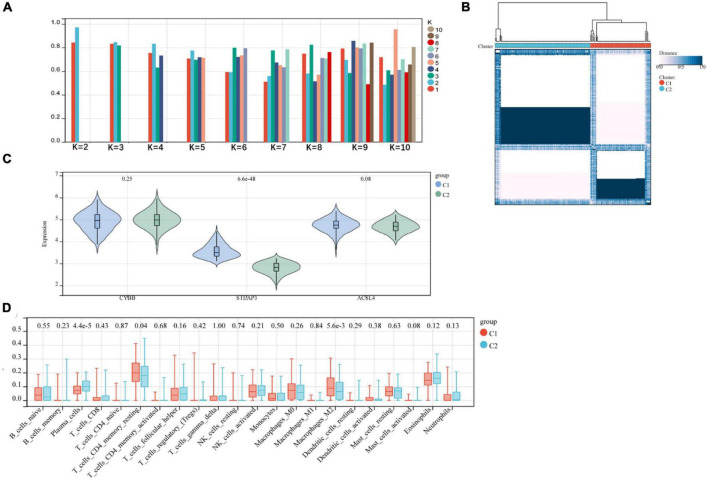
**(A,B)** Relevant candidate genes for consensus clustering analysis; **(C)** violin plot showing differential expression of relevant candidate genes between subgroups; **(D)** difference in immune infiltration between two subgroups.

We subjected the three relevant candidate gene models to consensus clustering (CC) analysis on the AD dataset GSE118553, considering intra-group consistency, and the highest number of clusters was assessed based on the average intra-group consistency of clusters, *K* = 2 (Figure 7A), and in the clustering heat map, we found that the clustering between different groups was most pronounced when *K* = 2 (Figure 7B), and was therefore divided into C1 and C2 two unsupervised clustering subgroups (Supplementary Table 1). The relevant candidate genes’ expression levels in the two subgroups were visualized by violin plots (Figure 7C), and significant variability was found for STEAP3 (*P* < 0.001). The amount of 22 immune cells in the two subgroups of AD was calculated by the Cibersort algorithm. The results showed statistically significant variability (*p* < 0.001) in Plasma Cells and Macrophages M2 between the two groups (Figure 7D).

### 3.7. Functional differences between various subgroups

**FIGURE 8:**
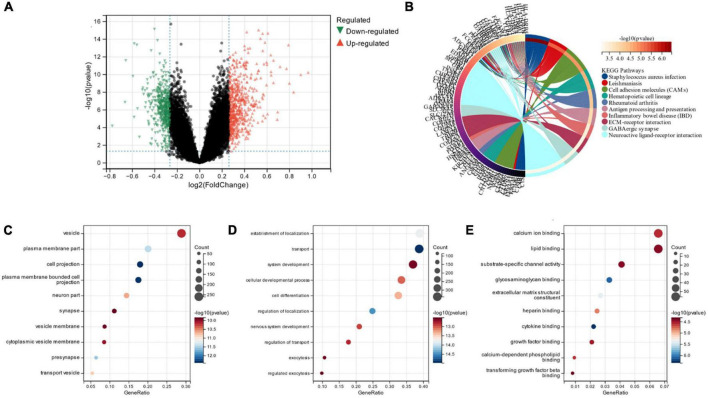
**(A)** Volcano map of subgroup DEG; **(B)** DEGs’ KEGG analysis; **(C)** DEGs’ GO analysis for cell composition; **(D)** DEGs’ GO analysis for biological processes; **(E)** DEGs’ GO analysis for molecular functions.

Limma analysis was conducted on the two subgroups, and a total of 1,119 DEGs were obtained. Out of these, 511 were down-regulated, and 608 were up-regulated (Figure 8A). We also performed FEA and KEGG analysis which revealed that the DEGs were chiefly located in the “vesicle” and “plasma membrane part” pathways (Figure 8B). GO analysis depicted that, in terms of cellular components, the DEGs were primarily located in the “vesicle” and “plasma membrane part” (Figure 8C). The chief biological processes associated with DEGs are “establishment of localization,” “transport” and “system development” (Figure 8D). MF analysis revealed that the most crucial items of DEGs were “lipid binding” and “calcium ion binding” (Figure 8E).

### 3.8. Gene–miRNA network diagram

**FIGURE 9:**
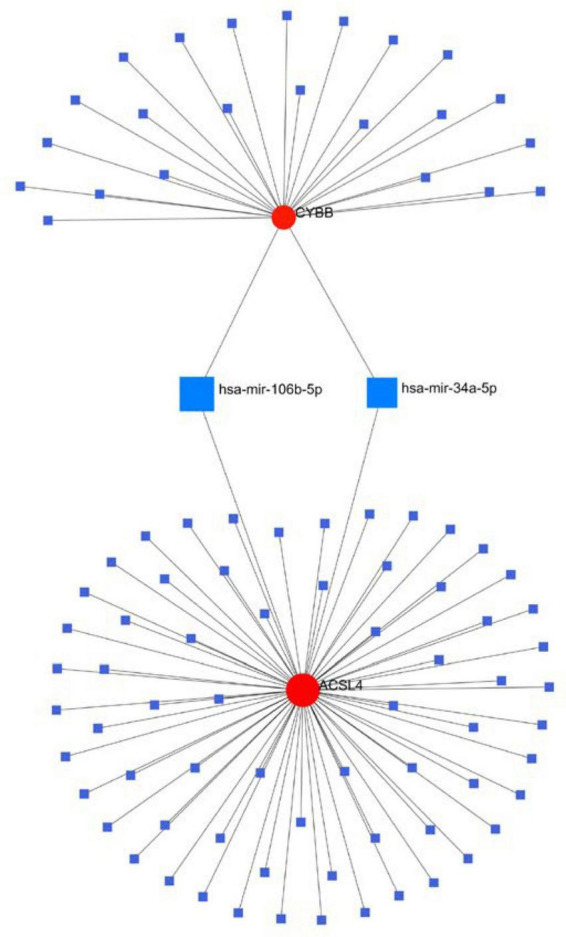
Interaction between candidate genes and miRNAs.

We generated the gene and miRNA-gene interaction networks by Networkanalyst. Three candidate gene-miRNA networks were constructed, and it was observed that hsa-mir-34a-5p and has-mir-106b-5p could regulate the expression of both CYBB and ACSL4 (Figure 9).

## 4. Discussion

Alzheimer’s disease is a progressive neurodegenerative disease that generally (>90%) presents in later life (65 + years) (Hersi et al., 2017). The huge majority of AD cases do not have a clear etiology, but various risk factors have been identified, such as female, obesity, heavy alcohol consumption, diabetes, and smoking (Schwarzinger et al., 2018). Numerous studies focused on the association between alcohol dependence and AD, and the mechanisms may be mainly related to the progressive accumulation of tau proteins, amyloid, age-dependent cognitive decline, accumulation of plaques and tangles, and age-dependent inflammation (Oddo et al., 2003; Janelsins et al., 2005; Belfiore et al., 2019).

Ferroptosis is a cell death mechanism driven by iron-mediated lipid peroxidation (Villalón-García et al., 2023). Neurodegenerative diseases arise from intricate neuronal cell death processes, which involve iron accumulation and lipid peroxidation in various brain regions (Guiney et al., 2017). Multiple studies have demonstrated that neuronal cell death often transpires due to ferroptosis (Ren et al., 2023; Wang D. et al., 2023). In our previous research, we have identified connections between psycho-neuro-degeneration and various factors, including programmed cell death (PCD), mitochondrial function, vesicular transport, and cuproptosis (Feng and Shen, 2023; Feng et al., 2023; Shen et al., 2023).

So what is the common link between AD, alcohol dependence, and Ferroptosis? In this study, we assessed the relationship between the three from a bioinformatics perspective. We obtained three target genes (CYBB, STEAP3, and ACSL4) and explained the possible mechanistic linkage through immune infiltration, functional enrichment analysis, machine learning algorithms, and consensus clustering analysis.

The primary immunodeficiency caused by mutations in the CYBB gene results in the inability of phagocytes to clear the infection (Wong et al., 2023). Numerous studies have demonstrated the role of CYBB as an inflammatory factor in developing multiple diseases (Chen et al., 2021; Hong et al., 2021; Wang et al., 2022).

STEAP3, a regulator strongly associated with Ferroptosis, affects several diseases through the immune infiltration pathway (Chen X. et al., 2023). STEAP3 is susceptible to m 6A-mediated family protein 2 of the YTH structural domain (YTHDF2), an N 6 -methyladenosine (m 6 A) reader, and is involved in the development of abnormal oxygen metabolism in the organism (Zhou et al., 2022).

Acyl-CoA Synthetase Long-Chain Family Member 4 (ACSL4) is a critical isozyme in polyunsaturated fatty acid (PUFA) metabolism (Zhou et al., 2023). ACSL4 would play a role in lipopolysaccharide (LPS)-induced microglia inflammation and has implications for diseases such as Parkinson’s disease and AD; however, the mechanism is not clear (Zhou et al., 2023). According to research by Wang M. E. et al. (2023), the direct E2F target gene ACSL4 appears to be essential for the sensitivity of RB1 loss-induced ferroptosis.

Micro RNA (miRNA) is a key regulator of disease gene expression. Numerous studies have demonstrated the extensive association between hsa-mir-34a-5p and AD (Li and Cai, 2021; Samadian et al., 2021), and studies by Alamro et al. (2022) demonstrated its use as a reliable indicator for diagnosing AD.

miR-106b-5p has the ability to inhibit the suppression of cell viability and certain DNA synthesis, thereby mediating cell death. Several studies have emphasized the importance of its involvement in coagulation, oxidative stress, and inflammatory pathways, particularly phosphorylation of tau, C-reactive protein (CRP), and neurofilaments, among others, showing its different plasma levels in AD patients (Mayo et al., 2021; Segaran et al., 2021).

With advances in molecular genetic phenotyping studies, the links between social behavior and genetic variation to protein and receptor function and disease development are being increasingly revealed. The findings of this study shed light on the possibility that alcohol addiction, as an exposure factor, may cause alterations in genetic markers of qtl, which in turn lead to differential expression of mRNA transcripts (CYBB, STEAP3 and ACSL4), which then further play a role in translational protein function (including, e.g., iron death in pcd and other metabolic pathways and alterations in the immune microenvironment) Such a line of research we think is interesting and we have been working for some time on a Mendelian randomization study analyzing neurodegenerative diseases and social behavior. Although this study has not yet fully elucidated the causal relationship, we believe that this step in the analysis of the mRNA and protein levels is important and meaningful.

## 5. Conclusion

In conclusion, we suggest that there is a link between AD, alcohol dependence, and Ferroptosis, which acts mainly through Ferroptosis-related metabolic pathways and the immune microenvironment, especially Regulatory T cells and gamma delta T cells. Three genes (CYBB, STEAP3, and ACSL4) and two miRNAs (hsa-mir-34a-5p and has-mir-106b-5p), which were mined on this basis, are strongly associated with these pathways. The diagnostic model built on the three genes was significant in predicting both AD and alcohol dependence.

## Data availability statement

The original contributions presented in this study are included in this article/Supplementary material, further inquiries can be directed to the corresponding author.

## Author contributions

MT, ZQ, YF, JS and PF wrote the main manuscript text. All authors reviewed the manuscript and approved the submitted version.

## Footnotes

1
www.ncbi.nlm.nih.gov/geo/

2
www.kegg.jp

3
org.Hs.eg.db

4
https://www.networkanalyst.ca/
